# Antenatal care surveillance for monitoring malaria prevalence and intervention coverage: a multicountry analysis

**DOI:** 10.1136/bmjgh-2024-018572

**Published:** 2025-09-30

**Authors:** Anna Munsey, Peder Digre, Joseph Hicks, Joseph Wagman, Molly Robertson, Manzidatou Alao, Aurore Ogouyemi Hounto, Adama Gansane, Siaka Debe, Baltazar Candrinho, Perpetua Uhomoibhi, Okefu Oyale Okoko, Ruth Lemwayi, Sijenunu Aron, Chabu C Kangale, Bupe M Kabamba, John Miller, Patrick Walker, Julie Gutman, ANC Surveillance Working Group, Kelly Davis

**Affiliations:** 1 Malaria Branch, Centers for Disease Control and Prevention, Atlanta, Georgia, USA; 2 PATH, Seattle, Washington, USA; 3 MRC Centre for Global Infectious Disease Analysis, School of Public Health, Imperial College London, London, UK; 4 PATH, Washington, District of Columbia, USA; 5 The Global Fund to Fight AIDS, Tuberculosis, and Malaria, Geneva, Switzerland; 6 Medical Care Development Global Health, U.S. Presidents’ Malaria Initiative Impact Malaria Project, Cotonou, Benin; 7 Unité de Parasitologie/Faculté des Sciences de la Santé, Université d’Abomey, Coutonou, Benin; 8 Centre National de Recherche et de Formation sur le Paludisme, Ouagadougou, Burkina Faso; 9 Research, Centre National de Recherche et de Formation sur le Paludisme, Ouagadougou, Burkina Faso; 10 National Malaria Control Programme, Mozambique Minister of Health, Maputo, Mozambique; 11 National Malaria Elimination Programme, Abuja, Nigeria; 12 Jhpiego, Dar es Salaam, Tanzania, United Republic of; 13 National Malaria Control Program, Dodoma, Tanzania, United Republic of; 14 PATH Programme for Advancing Malaria Outcomes, Lusaka, Zambia; 15 PATH Malaria Control and Elimination Partnership in Africa (MACEPA), Lusaka, Zambia

**Keywords:** Malaria, Epidemiology, Maternal health, Child health

## Abstract

Estimates of malaria prevalence and intervention coverage in Africa are primarily based on nationally representative household (HH) surveys. However, the expense and infrequency limit the utility of HH surveys for operational action by malaria programmes. We assessed whether data collected during first antenatal care (ANC1) visits, consisting of data on malaria prevalence using rapid diagnostic tests, ownership of insecticide-treated nets (ITNs) and treatment-seeking for children with fever, could provide relevant data to guide decision-makers. Malaria prevalence among ANC1 attendees in select areas of six countries (Benin, Burkina Faso, Mozambique, Nigeria, Tanzania and Zambia) was compared with prevalence data among children under 5 and school-aged children from cross-sectional HH surveys in the same areas. To examine the relationship between prevalence among ANC1 attendees and children, we fitted a linear trend to the log-OR of the risk of testing positive. The predictive performance of the model was assessed by leave-one-out cross-validation (LOOCV). District-level ANC1 prevalence and prevalence among children are correlated (Spearman’s rank correlation, r=0.79, 95% CI=0.65 to 0.88, p<0.001) and ANC1 prevalence is predictive of prevalence among children (LOOCV mean absolute error=6.5%). To understand whether data on ITN ownership collected at ANC1 are representative of ownership in the underlying communities, we assessed the district-level proportion of HH ownership in five countries (Benin, Burkina Faso, Mozambique, Nigeria and Zambia) and fitted an ordinal regression model to the ranking of ownership by district. Reported rates of treatment-seeking for children under 5 with fever, testing for malaria and treatment for the HH and ANC1 settings were compared. Estimates of malaria prevalence and ITN coverage derived from ANC1 attendees correlate well with HH survey estimates and may be useful in monitoring malaria prevalence and prevention efforts. In contrast, data on treatment-seeking does not appear useful.

**WHAT IS ALREADY KNOWN ON THIS TOPIC**

- The WHO has called for strengthening surveillance at all levels of malaria transmission. Prevalence of malaria among antenatal care (ANC) attendees has been shown to correlate with prevalence among children in the underlying communities in several settings if ANC attendees are tested regardless of symptoms. However, routine malaria testing at ANC is not performed in most malaria-endemic areas. There are limited data available on the value of administering questionnaires at ANC.

**WHAT THIS STUDY ADDS**

- We expand on earlier studies by detailing the relationship between prevalence among 36 018 ANC attendees and 25 442 children across a wide geographic scope and a range of malaria transmission intensities in six countries, and assess the representativeness of data on coverage of interventions collected from questionnaires administered at ANC. Prevalence at ANC is predictive of prevalence among children in the underlying community at the district level. Data collected on insecticide-treated net coverage is representative of that in the underlying communities, whereas data on treatment-seeking for children with fever appear subject to bias.

**HOW THIS STUDY MIGHT AFFECT RESEARCH, PRACTICE OR POLICY**

- Malaria control programmes and programmatic partners may consider implementing ANC surveillance and focused questionnaires as a means of tracking malaria burden and access to interventions in a timely, spatially granular manner, allowing data from this easy-to-access population to serve as a valuable complement to malaria indicator surveys.

## Introduction

 Since 2016, reductions in malaria burden have stalled, with the estimated global burden rising from 226 million cases in 2015 to 263 million cases in 2023.^1^ To accelerate progress towards malaria elimination, the WHO recommends strengthening surveillance at all levels of transmission intensity.^2^

Accurate and timely measurement of malaria burden is critical for appropriate resource allocation and assessment of intervention impact. Estimates of malaria burden calculated using case-based surveillance are hindered by uncertainty in catchment area population sizes, variability in seeking healthcare for fever and prevalence of other febrile illnesses.^3 4^ Thus, the majority of malaria cases and deaths in African countries are modelled estimates based on infection prevalence and intervention coverage obtained from household (HH) surveys.^5^ Due to their expense, HH surveys are conducted every 2–3 years and are powered only to the subnational administrative level. Such sparse data are of limited utility in programmatic decision-making at more granular levels.

Across sub-Saharan Africa, 83% of women achieve at least one antenatal (ANC) contact.^6^ If all women are tested for malaria irrespective of symptoms, malaria prevalence at ANC provides a more reliable measure of malaria burden, as it is less susceptible to biases introduced by trends in care-seeking behaviour and prevalence of other febrile diseases compared with case-based surveillance and provides significantly more spatial-temporal granularity compared with population-based surveys. Additionally, the opportunity to survey ANC attendees about coverage of malaria control interventions (eg, ownership of insecticide-treated nets, ITNs) may extend the utility of surveillance among this population. More evidence is needed on whether ANC attendees’ responses to surveys about malaria control interventions are representative of those of the underlying population.

Earlier studies have demonstrated malaria prevalence among pregnant women correlates with prevalence among under-5 (u5) children as well as with clinical malaria incidence, suggesting that data from ANC attendees may be used to track temporal trends, track progress in pre-elimination areas and to detect transmission hotspots.^7^,^10^ On average, prevalence in children tends to be higher than prevalence in pregnant women.^9^ However, primigravid women are at higher risk, and in pre-elimination settings, declines in ANC prevalence lag behind that of children.^5^ This study assessed whether data on malaria prevalence, ITN ownership and treatment seeking for children with fever obtained from women attending ANC in six countries—Benin, Burkina Faso, Mozambique, Nigeria, Tanzania and Zambia—are representative of prevalence among children, ITN ownership and treatment-seeking in the same areas. We expand on earlier studies by examining trends in malaria prevalence over a wide range of transmission intensities, including in areas in which seasonal malaria chemoprevention (SMC) is being conducted, and by assessing correlation of reported ITN ownership and treatment-seeking for febrile children between ANC surveys and community surveys in different settings. If validated, data from ANC could provide a valuable complement to malaria indicator surveys (MIS) and provide more meaningful data for allocating limited resources and assessing impact.

## Methods

### Study design

Data were collected from Benin, Burkina Faso, Mozambique, Nigeria, Tanzania and Zambia as part of three distinct parent studies. Each included: (1) collection of data from women attending ANC1 at selected health facilities, including results from malaria testing using a malaria rapid diagnostic test (mRDT) and a short questionnaire and (2) multiple cross-sectional HH surveys which consisted of testing children in the HH for malaria using an mRDT (regardless of symptoms at all study sites) and administering a questionnaire. The same questionnaire was used across all studies and settings and included questions on demographics and coverage of malaria control interventions including ownership of ITNs and treatment-seeking for febrile children. Maps of the study areas are shown in online supplemental figure 1. The study timeline for each country and dates of ITN campaigns are shown in online supplemental figure 2.

Across all studies, health facilities and HHs were selected at the second-level administrative area, which are named differently depending on the country (‘district’ in Benin, Burkina Faso, Mozambique and Zambia; ‘council’ in Tanzania; ‘local government area’ in Nigeria). For every country in the analysis, second-level administrative areas are collectively hereafter referred to as ‘districts’. The number of participants included in the analyses by study area during the cross-sectional surveys and at corresponding health facilities conducting ANC surveillance, and the number of participating health facilities per area, are listed in online supplemental table 1.

Data from Burkina Faso, Mozambique and Nigeria were collected during the New Nets Project (NNP), an observational study carried out between 2019 and 2022 to evaluate dual active-ingredient ITNs compared with pyrethroid-only nets at reducing malaria transmission.^11^ In each country, districts included in the NNP study were selected based on geographic proximity and comparability in malaria transmission and consist of three districts in Burkina Faso, three districts in Mozambique and four districts in Nigeria. In each district in Burkina Faso and Mozambique, seven health facilities conducting ANC services with a mean of at least 20 ANC1 attendees per month were selected. In each district in Nigeria, 10 health facilities with the highest average number of ANC1 attendees per month were selected. Cross-sectional surveys were conducted prior to or during ITN distribution campaigns and during subsequent study years. Details on selection of HHs and selection of children for parasitaemia testing within HHs can be found in the NNP Final Report.^11^ Across all NNP study HHs, one child between the ages of 6 and 59 months was selected at random for testing and the head of HH or primary caregiver responded to the questionnaire.

In Burkina Faso, the switch from four to five cycles of SMC in the study districts resulted in overlap in timing with the NNP surveys, with children aged 3–59 months already receiving the benefit of the first round of SMC at the time of HH surveys. Therefore, in Burkina Faso, one child aged 5–10 years per HH was randomly selected for testing and included in the analyses. In Nigeria, Asa and Moro implemented SMC beginning in 2021. One child per HH aged 5–15 years was selected at random for testing in all study districts in Nigeria in 2021 and 2022 and included in the analyses. In each country, children who had already received SMC are not included in the analyses of prevalence.

In Tanzania and Benin, data were collected in the context of cluster randomised trials of group ANC in all six districts in Geita Region, northwest Tanzania, and in all eight districts in Atlantique Department, Benin.^12^ Tanzania began implementing malaria screening and treatment for all ANC1 attendees in 2014; monthly aggregated mRDT positivity data derived from routine malaria testing conducted at ANC1 were obtained from District Health Information Software version 2. In this system, ANC1 prevalence data are stratified into women less than 20 years of age and women equal to or greater than 20 years of age. Neither exact age nor gravidity is available from the data used in this analysis. In Tanzania and Benin, 40 facilities with an average monthly ANC1 attendance of 20–120 women and which met selection criteria to conduct an intervention trial of group ANC were selected. Cross-sectional HH surveys were conducted in randomly selected HHs within each included health facility catchment area. In selected HHs, an mRDT was performed for all children aged 6–59 months in selected HHs. Additional details on study design for Tanzania and Benin can be found in Munsey *et al* and Ochieng *et al*.^10 12^ (SMC is implemented in some areas of Benin, though it is not currently implemented in Atlantique.)

Data from Zambia were collected alongside the Proactive Community Case Management for Malaria (ProCCM) study, a 3-year cluster-randomised controlled trial in Chadiza District to assess the impact of weekly HH visits by community health workers (CHWs).^13^ Within Chadiza, routine malaria testing at ANC1 was integrated in the health facilities providing ANC services. In the 18 health facility catchment areas included in the study, all HHs within randomised CHW catchment areas were selected for the cross-sectional surveys. Heads of HHs responded to the questionnaire and all HH members present were tested for malaria. Only prevalence data from children aged 6 to 59 months are included in the analyses. Additional details on the ProCCM study design can be found in Rutagwera *et al*.^13^

### Parasitaemia

To quantify the relationship between risk of testing positive by mRDT in children versus ANC attendees across districts, and to assess whether this varies by transmission level, we fitted a linear trend to the log-odds of the risk of testing positive by mRDT following the approach of Okell *et al*.^14^ Three Bayesian mixed effects models were fitted separately: (1) all women, (2) women under 20 years of age (Tanzania) or primigravida women (all other countries) and (3) women 20 years of age and older (Tanzania) or secundigravida and multigravida (hereafter collectively referred to as ‘multigravida’; all other countries). A district-level random effect term was included; see online supplemental methods for details on model fitting. To assess the predictive performance of the models, leave-one-out cross-validation (LOOCV)^15^ was performed, separately assessing the performance of the models with all women, and disaggregating by age/gravida. The mean absolute error (MAE) of observed vs predicted district-level prevalence for children was calculated. For descriptive purposes, study site transmission intensities were categorised relative to one another as follows: (1) low transmission (prevalence among children <20%), (2) moderate transmission (prevalence among children 20%–25%) or (3) high transmission (prevalence among children >25%).

### ITN ownership

Within Burkina Faso, Benin, Mozambique, Nigeria and Zambia, the proportion of ANC1 attendees and cross-sectional survey respondents reporting ITN ownership at the level of one ITN per HH and one ITN per two persons was calculated for each survey. Spearman’s rank correlation was used to determine correlation between ownership reported at ANC and in cross-sectional surveys. Each district survey time point was ranked (ie, from highest ITN ownership to lowest ITN ownership), and an ordinal regression model was used to determine if the rank of district-level ownership as determined from ANC1 questionnaires is predictive of the rank of district-level ownership as reported in HH cross-sectional surveys. See online supplemental methods for details on model fitting.

### Treatment-seeking for children with fever

ANC1 attendees and HH survey respondents who reported a fever among one or more children under 5 years in their HH within the last 2 weeks were asked whether they sought treatment for the child, and if so, whether the child was tested for malaria and whether any medication was received. Reported (1) percentages of treatment-seeking for fever, (2) percentages testing for malaria among those who sought treatment and (3) percentages of receiving any medication among those who sought treatment were calculated for ANC1 attendees, aggregated across all study sites and timepoints, and separately for HH survey respondents, aggregated across all study sites and time points. ORs for the three reported metrics were calculated (odds of affirmative response at ANC1/odds of affirmative response at HH).

### Patient and public involvement

Patients were not involved in setting the research questions, outcome measures or design of the studies. The burden of participation in the studies is thought to be outweighed by the benefit of participation, that is, the understanding of one’s malaria status and the receipt of anti-malarial medications if indicated.

## Results

Health facilities included in the study tested a median of 27 women (range 6–223) per month, totalling a median of 171 women per district per month (range 30 – 958).

### Parasitaemia

Transmission intensities ranged from low (Geita Town, Tanzania: mean prevalence at HH surveys=8%, 95% CI 5.3% to 11.5%; Changara and Guro, Mozambique; mean prevalence at HH surveys=4.9%, 95% CI 3.5% to 6.5%; 5.5%, 95% CI 4% to 7.2% respectively) to high (Asa, Nigeria; mean prevalence at cross-sectional surveys=50.1%, 95% CI 47.4% to 52.8%). At the country level, the highest prevalence at both ANC1 and HH was in Nigeria (40.9%, 95% CI 39% to 42.7%; 44.5%, 95% CI 43.4% to 45.7%, respectively). Prevalence by country and group is shown in table 1; these data are disaggregated by district and study year in online supplemental table 2. Among countries conducting SMC (Burkina Faso and Nigeria), prevalence by age group is shown in online supplemental tables 3a–4d. Among children tested in cross-sectional HH surveys and women attending ANC1 of all ages/gravida in selected months (online supplemental figure 2), Spearman’s correlation was 0.79 (95% CI 0.65 to 0.88, p<0.001). Among districts conducting SMC, Spearman’s correlation was 0.69 (95% CI 0.05 to 0.94%, p=0.016).

**Table 1:** Number of children or women tested, mean prevalence and 95% CIs across all study years by country and group

	Children		ANC1—all women		ANC1—multigravida		ANC1—primigravid	
	N	Prevalence (95% CI)	N	Prevalence (95% CI)	N	Prevalence (95% CI)	N	Prevalence (95% CI)
Benin	4458	29.2 (27.9 to 30.5)	4621	25.5 (24.2 to 26.8)	3515	23.3 (21.9 to 24.8)	1106	32.4 (29.6 to 35.2)
Burkina Faso	1239	37.6 (34.9 to 40.4)	3318	24.7 (23.2 to 26.2)	2478	20.1 (18.6 to 21.8)	840	38.1 (34.8 to 41.5)
Mozambique	2523	17.2 (15.7 to 18.7)	5702	13.1 (12.2 to 14)	4122	11.5 (10.5 to 12.5)	1580	17.3 (15.5 to 19.3)
Nigeria	6902	44.5 (43.4 to 45.7)	2839	40.9 (39 to 42.7)	1933	39.1 (36.9 to 41.3)	906	44.6 (41.3 to 47.9)
Tanzania	6300	23.5 (22.5 to 24.6)	17 818	15.8 (15.2 to 16.3)	14 527	13.2 (12.6 to 13.8)	3291	27.2 (25.7 to 28.8)
Zambia	4020	18.2 (17 to 19.4)	1720	19.3 (17.5 to 21.2)	1199	16.4 (14.4 to 18.7)	521	25.9 (22.2 to 29.9)

ANC1 data include only the months selected for inclusion in the analyses.

ANC, antenatal care.

**Figure 1:**
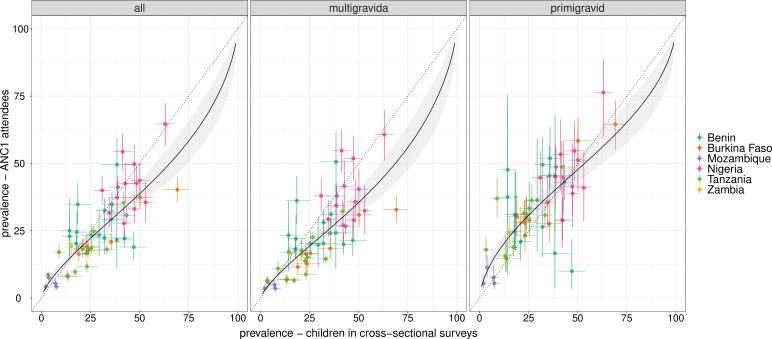
Fitted models of prevalence among children at cross-sectional household (HH) surveys vs. first antenatal care (ANC1) attendees by ANC attendee gravida/age category. District level data with 95% credible intervals are shown, coloured by country. ANC, antenatal care.

Fitted models of prevalence at the district level are shown in figure 1. In the low and moderate transmission settings (Mozambique, Tanzania, Zambia), there are approximately linear relationships between prevalence among all gravida and multigravida women and children (as evidenced by the slope of the best-fitting regression lines closely approximating the identity lines), whereas prevalence among primigravid women is higher than that of children. In the high transmission settings (Benin, Burkina Faso, Nigeria), there is more heterogeneity in ANC prevalence, with the relationship between prevalence among primigravid and children being closer to linear and multigravida tending to have lower prevalence relative to the other groups. In the LOOCV models, MAE between predicted and observed prevalence among children was 6.5% using all gravida, 8.7% using multigravida only and 6.8% using primigravid only (online supplemental figures 3a–c).

### ITN ownership

Across all study sites and timepoints, ITN coverage of one ITN per HH was 80.0% (95% CI 79.3% to 80.7%) at ANC1 and 73.2% (95% CI 72.5% to 73.9%) in HH surveys. At the level of one ITN per two persons, coverage was 43.2% (95% CI 42.3% to 44.0%) at ANC1 and 30.1% (95% CI 29.4% to 30.8%) in HH surveys. By both coverage metrics and settings, Benin had the highest ITN coverage (one ITN=94.6%, 95% CI 93.8% to 95.3% at ANC1; one ITN per two persons=50.1%, 95% CI 49.3% to 52.6% at ANC1), while Zambia had the lowest by both coverage metrics and settings (one ITN=66.2%, 95% CI 63.9% to 68.5% at ANC1; one ITN per two persons=22.0%, 95% CI 20.1% to 24.1% at ANC1). ITN ownership by district and group is shown in online supplemental tables 5 and 6.

At the level of one ITN per HH, there was a strong district-level correlation between ANC surveys and cross-sectional surveys (r=0.63, 95% CI 0.33 to 0.83, p<0.001). At the level of one ITN per two persons, there was moderate correlation (r=0.50, 95% CI 0.19 to 0.73, p=0.002). In the ordinal regression models, district-level rank of ITN ownership as assessed at ANC1 was predictive of rank of ITN ownership in the cross-sectional surveys at both levels of coverage (one net per HH ANC1 rank regression coefficient=0.35, 95% CI=0.22 to 0.49; one net per two persons ANC1 rank regression coefficient=0.3, 95% CI=0.17 to 0.42).

### Treatment-seeking for children with fever

Data were collected on 7135 fevers at ANC and 2610 fevers in HH surveys. Data on number of fevers and rates of treatment seeking, testing and treatment by setting and country are shown in online supplemental table 7. In all six countries, reported rates of treatment-seeking for children with fever were higher at ANC1 (83.1%, 95% CI 82.2% to 84.0%) than in HH surveys (66.2%, 95% CI 64.3% to 68.0%; OR, for treatment-seeking among ANC1 attendees=2.5, 95% CI 2.2 to 2.7). ANC1 attendees were also more likely to report the child had blood drawn for testing and the child had received any treatment (OR for testing among ANC1 attendees=3.5, 95% CI 3.1 to 3.9; OR for treatment=2.3, 95% CI 1.9 to 2.7).

## Discussion

In addition to the clinical benefit for each individual woman in testing for malaria at ANC1,^16 17^ ANC1 surveillance can provide continuous, more spatially granular data on parasitaemia prevalence. In this study, we add evidence to the growing body of literature which suggests ANC attendees can serve as a valuable population for surveillance and better characterise how transmission levels affect the relationship between malaria prevalence among pregnant women and that among children.

Across the spectrum of transmission intensities, primigravid women tend to have higher prevalence than multigravida women, as they have not yet acquired pregnancy-specific immunity.^18^ Primigravid women may also be less likely to use an ITN prior to attending ANC1 as compared with multigravida women who previously received an ITN from ANC distribution, increasing their risk of exposure.^10 19^

In low-transmission settings, reductions in prevalence among pregnant women lag behind that of children, allowing ANC1 attendees to serve as an especially valuable population for tracking progress toward elimination in these settings.^5^ This was supported in our findings; in Geita Town, Tanzania, prevalence among children at baseline was 3.4% (95% CI 1.2% to 7.2%), whereas prevalence among ANC1 attendees was 8.6% (95% CI 7.4% to 9.9%). There were similar findings in Changara and Guro, Mozambique.

In high-transmission settings, women are relatively more likely to be exposed and thus acquire pregnancy-specific immunity during their first pregnancy. On subsequent pregnancies, these women will have parasite densities below the detection limit of mRDTs (800 pg/mL of HRP2 antigen).^20^,^22^ As a result, prevalence among children tends to exceed that of multigravida women in high-transmission areas, whereas the relationship between prevalence in children and primigravid women tends to be more linear in high-transmission settings.^5 8 9 23^ In general, there is more heterogeneity in ANC prevalence at higher-transmission settings.^9^ Despite this, our models demonstrated good predictive performance, with the model including all women having the best performance, followed by the model including only primigravid women. Additionally, the strong correlation demonstrated between prevalence at ANC1 and among children was maintained in the areas conducting SMC, though this estimate is less precise, owing to fewer datapoints available for analysis. The ability to capture the location of the women’s residence could further improve the utility of these data, particularly in areas where women may not attend ANC at their nearest health facility, allowing tracking of trends at a finer spatial granularity.

The opportunity to survey women attending ANC1 may provide additional useful information for malaria control programmes on access and utilisation of interventions. Questionnaires administered in this study were modelled after those used for Demographic and Health Surveys and MIS. We analysed data collected via questionnaires administered at ANC and demonstrated these data can track trends in ITN ownership. If ITN ownership remains universally high within a survey region, ANC questionnaires are unlikely to capture minor heterogeneities in time or space.^10^ However, several mass ITN campaigns within the study areas allowed for sufficient variation such that ANC data were able to predict ownership in the community by district across multiple time points, highlighting how these data could be used to prioritise areas which may benefit from periodic campaigns to increase existing ITN ownership.

Responses about treatment-seeking for children with fever in the health facility setting appear to be biased, with ANC attendees more likely to report treatment-seeking, testing for malaria and treatment relative to those surveyed in HHs. These results suggest assessment of treatment-seeking behaviours at ANC is not reliable, with social desirability bias likely influencing the responses of those surveyed in the clinical setting more than those surveyed at home.

It is crucial to understand the potential community-level and programmatic implications of these data, as well as their limitations. Overall, prevalence among ANC1 attendees as assessed by mRDT may underestimate malaria prevalence among the entire population of pregnant women, as (1) the parasite tends to sequester in the placenta, reducing diagnostic sensitivity especially among multigravida^8 20 24 25^ and (2) rural women and women of lower socioeconomic status are less likely to attend ANC but are at higher risk for malaria.^26^,^30^ This may be less relevant in countries with very high ANC attendance, but is an especially important consideration in Nigeria, which had the highest prevalence in this study but has low ANC attendance rates relative to other malaria-endemic countries.^31 32^ Focused community-based surveys which assess prevalence among pregnant women may help determine the risk among those women who do not attend ANC.

While this study was not specifically designed to assess the utility of ANC1 surveillance in areas conducting SMC, our results suggest ANC1 parasitaemia correlates with parasitaemia among children who have not received SMC (mostly older children in this study), demonstrating ANC1 data may provide stable, robust estimates of community-level malaria trends in the face of SMC. Further work, for example, analysing longitudinal ANC data in SMC areas, may help to elucidate the knock-on effects of SMC on age groups which are not directly receiving chemoprevention.^33 34^

Staffing shortages and costs of additional mRDTs and artemisinin-based combination therapies have been reported as barriers to screen-and-treat methods at ANC compared with intermittent preventative treatment alone.^5 16 17^ Data on improved outcomes, such as prevalence of low birth weight, may provide support for scale-up of malaria testing at ANC1. The further addition of questionnaires at ANC, even if conducted at infrequent cadences, would need to carefully consider their utility against added costs, feasibility, acceptability among staff and risk of compromising other ANC services.

## Conclusions

If ANC1 attendees are routinely tested regardless of symptoms, ANC data have clear advantages over clinical incidence data from outpatient facility settings, including the ability to use data from ANC to separate trends in clinical incidence from trends in other febrile illnesses and treatment-seeking,^5 7 35^ in addition to providing benefit to the individual woman. The ability to track prevalence at second-level administrative areas represents improvements over MIS and is likely sufficient for tracking the impact of interventions. The opportunity to survey ANC attendees regarding ITN ownership may extend the utility of data which could be collected from this easily accessible population, whereas responses related to treatment-seeking are prone to bias.

## Supplementary material

10.1136/bmjgh-2024-018572 online supplemental file 1

## Data Availability

Data from this study can be accessed upon request through the corresponding author, who will submit a formal request to the Ministries of Health.
