# Supplementary material for: Antenatal care surveillance for monitoring malaria prevalence and intervention coverage: a multicountry analysis
Source: BMJ Glob Health. 2025 Sep 30;10(9):e018572. doi: 10.1136/bmjgh-2024-018572 (PMC12496046; doi:10.1136/bmjgh-2024-018572)
Supplement: online supplemental file 1 [file bmjgh-10-9-s001.docx]

**Supplement to:** **Antenatal care surveillance for monitoring prevalence and intervention coverage—a multi-country analysis**

Anna Munsey^1^, Peder Digre^2^, Joseph Hicks^3^, Joseph Wagman^4^, Molly Robertson^5^, Manzidatou Alao^6^, Aurore Ogouyemi Hounto^7^, Adama Gansane^8^, Siaka Debe^8^, Baltazar Candrinho^9^, Perpetua Uhomoibhi^10^, Okefu Oyale Okoko^10^, Ruth Lemwayi^11^, Sijenunu Aaron^12^, Chabu Kangale^13^, Bupe Kabamba^13^, John Miller^13^, Patrick G.T. Walker^3^, Julie R. Gutman^1^, ANC Surveillance Working Group*

1 *Malaria Branch, Division of Parasitic Diseases and Malaria, National Center for Emerging and Infectious Zoonotic Diseases, Centers for Disease Control and Prevention, Atlanta GA, USA*; 2 *PATH, Seattle, WA, USA*; 3 *MRC Centre for Global Infectious Disease Analysis, School of Public Health, Imperial College, London, UK*; 4 *PATH, Washington, DC, USA*; 5 *The Global Fund to Fight AIDS, Tuberculosis, and Malaria, Geneva, Switzerland;* 6 *U.S. Presidents’ Malaria Initiative Impact Malaria Project, Medical Care Development Global Health, Cotonou, Benin*; 7 *Unité de Parasitologie/Faculté des Sciences de la Santé, Université d’Abomey, Calavi, Cotonou, Benin*; 8 *Centre National de Recherche et Formation sur le Paludisme, Ouagadougou, Burkina Faso;* 9 *National Malaria Control Program, Ministry of Health, Maputo, Mozambique*; 10 *National Malaria Elimination Programme, Abuja, Nigeria*; 11 *Jhpiego, Dar es Salaam, Tanzania*; 12 *Ministry of Health, Tanzania*; 13 *PATH, Lusaka, Zambia*

**Membership of the ANC Surveillance Working Group is provided in the Acknowledgments of the main text.*

Contents

[Supplementary Methods 3](#_Toc183426372)

[References 4](#_Toc183426373)

[Supplementary Figures 5](#_Toc183426374)

[Supplementary Figure 1. Locations of the six countries (Burkina Faso, Benin, Nigeria, Tanzania, Zambia, and Mozambique) within Africa. Study districts are shaded in yellow. 5](#_Toc183426375)

[Supplementary Figure 2. Study timelines indicating the dates of cross-sectional household surveys and months of ANC surveillance data selected for inclusion in models. 6](#_Toc183426376)

[Supplementary Figure 3a. Predicted vs. observed test positivity rates among children from the leave-one-out cross-validation models using data from all women, colored by country. 7](#_Toc183426377)

[Supplementary Figure 3b. Predicted vs. observed test positivity rates among children from the leave-one-out cross-validation models using data from multigravida women, colored by country. 7](#_Toc183426378)

[Supplementary Figure 3c. Predicted vs. observed test positivity rates among children from the leave-one-out cross-validation models using data from primigravid women, colored by country. 8](#_Toc183426379)

[Supplementary Tables 9](#_Toc183426380)

[Supplementary Table 1 9](#_Toc183426381)

[Supplementary Table 2 10](#_Toc183426382)

[Supplementary Tables 3a-d. TPRs by age group, Nigeria, 2021 (3a-b) and 2022 (3c-d). 11](#_Toc183426383)

[Supplementary Tables 4a-d. TPRs by age group, Burkina Faso, 2021 (4a-b) and 2022 (4c-d). 12](#_Toc183426384)

[Supplementary Table 5 13](#_Toc183426385)

[Supplementary Table 6 14](#_Toc183426386)

[Supplementary Table 7. 15](#_Toc183426387)

## Supplementary Methods

Parasitemia models

The relationship between parasitemia among ANC1 attendees and children in the same second-level administrative area (“site”) was assessed by fitting a linear trend to the log-odds ratio (OR) of the risk of testing positive by mRDT, wherein the probability of testing positive (*p*) at each site *i* was modeled as a binomial distribution:

y_c,i_ ~ binomial(p*_c,i_,* n*_c,i_*),

logit*(*p*_p,i_) = θ_c,i_ +*$\delta_{i}$*,*

$$\delta_{i}= \beta_{0}\theta_{c,i}+ \theta_{p,i}+\tau$$

where *p_c,i_* is the probability of children at site *i* being test-positive, *p_p,i_* is the probability of ANC1 attendees being test-positive, *θ_c,i_* and *θ_p,i_* are equivalent log odds of being test-positive,$\delta_{i}$ is the log odds ratio (OR) of testing positive, τ is the site-level random effects, and *β*_0_ is the regression coefficient assessing whether the relationship varies by underlying prevalence in childhood. The models were fitted using Bayesian Markov Chain Monte Carlo (MCMC) in R 4.4.0 [1] using R2OpenBUGS [2] with 50,000 iterations each. Convergence of parameter estimates was verified with Helman-Rubin R-hat values [3].

Due to variation in design of the parent studies, the number of months of included ANC data and their temporal position relative to the cross-sectional surveys vary slightly by location. ANC data were selected from available data to provide the most temporal overlap with the household surveys (Supplementary Figure 2).

ITN models

Although Tanzania conducts routine ANC1 parasitemia surveillance, questionnaires are not administered to ANC attendees as part of the routine system. Due to budgetary constraints, there was a gap ranging from one to four months between cross-sectional surveys and administering of questionnaires at ANC; thus, ITN coverage data from Tanzania are not included in the analysis presented here. An analysis of ITN ownership in Geita Region is presented in Munsey et al. 2023 [4]. Similarly, in Benin, there were no overlapping dates of ANC questionnaires available for comparison to cross-sectional surveys from Abomey-calavi (baseline and end line) and So-ava (baseline). For the remaining districts, ordinal regression (cumulative probit) models were fitted using Bayesian MCMC in R 4.2.2 [5] using the brms package [6-8]. Noninformative priors were used for all model parameters. Country was included as a random effect term. Each model was run with four chains for 2,000 iterations within a burn-in period of 1,000. Convergence of parameter estimates was verified with Helman-Rubin R-hat values [3]. 95% CIs represent Bayesian credible intervals.

## References

1. R Core Team, *R: A Language and Environment for Statistical Computing*. 2024, R Foundation for Statistical Computing.

2. Sturtz, S.L., Uwe; Gelman, Andrew, *R2WinBUGS: A Package for Running WinBUGS from R.* Journal of Statistical Software, 2005. **12**: p. 1-16.

3. Gelman, A., et al., *Bayesian Data Analysis, Third Edition*. 2013: Taylor & Francis.

4. Munsey, A., et al., *Assessing the utility of pregnant women as a sentinel surveillance population for malaria in Geita, Tanzania, 2019 - 2021.* Int J Infect Dis, 2023. **136**: p. 57-63.

5. Team, R.C., *R: A Language and Environment for Statistical Computing*. 2022, R Foundation for Statistical Computing.

6. Bürkner, P.-C., *brms: An R Package for Bayesian Multilevel Models Using Stan.* Journal of Statistical Software, 2017. **80**(1).

7. Bürkner, P.-C., *Advanced Bayesian Multilevel Modeling with the R Package brms.* The R Journal, 2018. **10**(1).

8. Bürkner, P.-C., *Bayesian Item Response Modeling in R with brms and Stan.* Journal of Statistical Software, 2021. **100**(5).

## Supplementary Figures


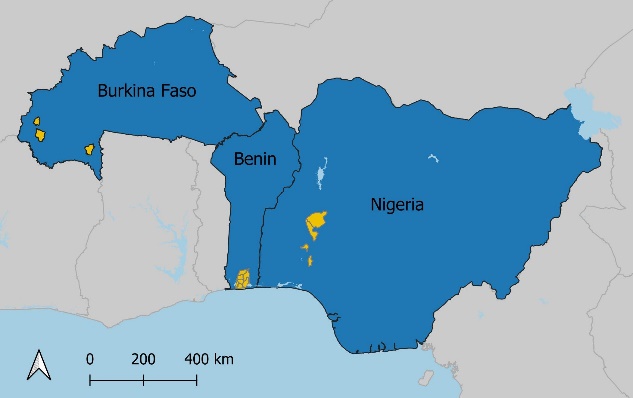

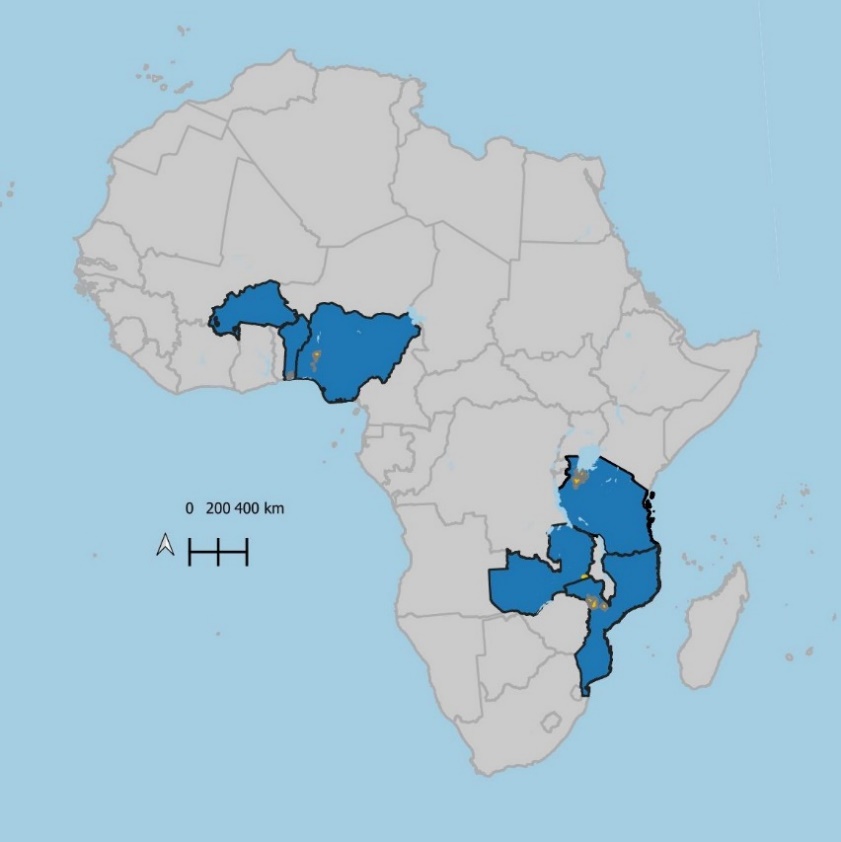


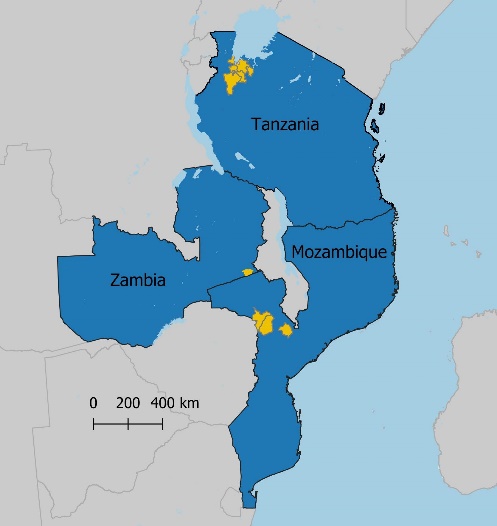


## Supplementary Figure 1. Locations of the six countries (Burkina Faso, Benin, Nigeria, Tanzania, Zambia, and Mozambique) within Africa. Study districts are shaded in yellow.

|  | 2019 | | | | | | | | | | | | 2020 | | | | | | | | | | | | 2021 | | | | | | | | | | | | | |
| --- | --- | --- | --- | --- | --- | --- | --- | --- | --- | --- | --- | --- | --- | --- | --- | --- | --- | --- | --- | --- | --- | --- | --- | --- | --- | --- | --- | --- | --- | --- | --- | --- | --- | --- | --- | --- | --- | --- |
|  | J | F | M | A | M | J | J | A | S | O | N | D | J | F | M | A | M | J | J | A | S | O | N | D | J | F | M | A | M | J | | J | A | S | O | N | D |  |
| Benin |  |  |  |  |  |  |  |  |  |  |  |  |  |  |  |  |  |  |  |  |  |  |  |  |  |  |  |  |  |  | |  |  |  |  |  |  |  |
|  |  |  |  |  |  |  |  |  |  |  |  |  |  |  |  |  |  |  |  |  |  |  |  |  |  |  |  |  |  |  | |  |  |  |  |  |  |  |
|  |  |  |  |  |  |  |  |  |  |  |  |  |  |  |  |  |  |  |  |  |  |  |  |  |  |  |  |  |  |  | |  |  |  |  |  |  |  |
| Burkina Faso |  |  |  |  |  |  |  |  |  |  |  |  |  |  |  |  |  |  |  |  |  |  |  |  |  |  |  |  |  |  | |  |  |  |  |  |  |  |
|  |  |  |  |  |  |  |  |  |  |  |  |  |  |  |  |  |  |  |  |  |  |  |  |  |  |  |  |  |  |  | |  |  |  |  |  |  |  |
|  |  |  |  |  |  |  |  |  |  |  |  |  |  |  |  |  |  |  |  |  |  |  |  |  |  |  |  |  |  |  | |  |  |  |  |  |  |  |
| Mozambique |  |  |  |  |  |  |  |  |  |  |  |  |  |  |  |  |  |  |  |  |  |  |  |  |  |  |  |  |  |  | |  |  |  |  |  |  |  |
|  |  |  |  |  |  |  |  |  |  |  |  |  |  |  |  |  |  |  |  |  |  |  |  |  |  |  |  |  |  |  | |  |  |  |  |  |  |  |
|  |  |  |  |  |  |  |  |  |  |  |  |  |  |  |  |  |  |  |  |  |  |  |  |  |  |  |  |  |  |  | |  |  |  |  |  |  |  |
| Nigeria |  |  |  |  |  |  |  |  |  |  |  |  |  |  |  |  |  |  |  |  |  |  |  |  |  |  |  |  |  |  | |  |  |  |  |  |  |  |
|  |  |  |  |  |  |  |  |  |  |  |  |  |  |  |  |  |  |  |  |  |  |  |  |  |  |  |  |  |  |  | |  |  |  |  |  |  |  |
|  |  |  |  |  |  |  |  |  |  |  |  |  |  |  |  |  |  |  |  |  |  |  |  |  |  |  |  |  |  |  | |  |  |  |  |  |  |  |
| Tanzania |  |  |  |  |  |  |  |  |  |  |  |  |  |  |  |  |  |  |  |  |  |  |  |  |  |  |  |  |  |  | |  |  |  |  |  |  |  |
|  |  |  |  |  |  |  |  |  |  |  |  |  |  |  |  |  |  |  |  |  |  |  |  |  |  |  |  |  |  |  | |  |  |  |  |  |  |  |
|  |  |  |  |  |  |  |  |  |  |  |  |  |  |  |  |  |  |  |  |  |  |  |  |  |  |  |  |  |  |  | |  |  |  |  |  |  |  |
| Zambia |  |  |  |  |  |  |  |  |  |  |  |  |  |  |  |  |  |  |  |  |  |  |  |  |  |  |  |  |  |  | |  |  |  |  |  |  |  |
|  |  |  |  |  |  |  |  |  |  |  |  |  |  |  |  |  |  |  |  |  |  |  |  |  |  |  |  |  |  |  | |  |  |  |  |  |  |  |
|  |  |  |  |  |  |  |  |  |  |  |  |  |  |  |  |  |  |  |  |  |  |  |  |  |  |  |  |  |  |  | |  |  |  |  |  |  |  |
|  | 2022 | | | | | | | | | | | | 2023 | | | | | | | | | | | |  |  |  |  |  | |  |  |  |  | | | | |
|  | J | F | M | A | M | J | J | A | S | O | N | D | J | F | M | A | M | J | J | A | S | O | N | D |  |  |  |  |  | |  |  |  |  | | | |  |
| Benin |  |  |  |  |  |  |  |  |  |  |  |  |  |  |  |  |  |  |  |  |  |  |  |  |  |  |  |  |  | |  |  |  |  | | | |  |
|  |  |  |  |  |  |  |  |  |  |  |  |  |  |  |  |  |  |  |  |  |  |  |  |  |  |  |  |  |  | |  |  |  |  | | | |  |
|  |  |  |  |  |  |  |  |  |  |  |  |  |  |  |  |  |  |  |  |  |  |  |  |  |  |  |  |  |  | |  |  |  |  | | | |  |
| Burkina Faso |  |  |  |  |  |  |  |  |  |  |  |  |  |  |  |  |  |  |  |  |  |  |  |  |  |  |  |  | Cross-sectional survey | | | | | | | | |  |
|  |  |  |  |  |  |  |  |  |  |  |  |  |  |  |  |  |  |  |  |  |  |  |  |  |  |  |  |  |  | |  |  |  |  | | | |  |
|  |  |  |  |  |  |  |  |  |  |  |  |  |  |  |  |  |  |  |  |  |  |  |  |  |  |  |  |  |  | |  |  |  |  | | | |  |
| Mozambique |  |  |  |  |  |  |  |  |  |  |  |  |  |  |  |  |  |  |  |  |  |  |  |  |  |  |  |  | ANC survey | | | | |  | | | |  |
|  |  |  |  |  |  |  |  |  |  |  |  |  |  |  |  |  |  |  |  |  |  |  |  |  |  |  |  |  |  | |  |  |  |  | | | |  |
|  |  |  |  |  |  |  |  |  |  |  |  |  |  |  |  |  |  |  |  |  |  |  |  |  |  |  |  |  |  | |  |  |  |  | | | |  |
| Nigeria |  |  |  |  |  |  |  |  |  |  |  |  |  |  |  |  |  |  |  |  |  |  |  |  |  |  |  |  | ITN campaign | | | | | | | | |  |
|  |  |  |  |  |  |  |  |  |  |  |  |  |  |  |  |  |  |  |  |  |  |  |  |  |  |  |  |  |  | |  |  |  |  | | | |  |
|  |  |  |  |  |  |  |  |  |  |  |  |  |  |  |  |  |  |  |  |  |  |  |  |  |  |  |  |  |  | |  |  |  |  | | | |  |
| Tanzania |  |  |  |  |  |  |  |  |  |  |  |  |  |  |  |  |  |  |  |  |  |  |  |  |  |  |  |  |  | |  |  |  |  | | | |  |
|  |  |  |  |  |  |  |  |  |  |  |  |  |  |  |  |  |  |  |  |  |  |  |  |  |  |  |  |  |  | |  |  |  |  | | | |  |
|  |  |  |  |  |  |  |  |  |  |  |  |  |  |  |  |  |  |  |  |  |  |  |  |  |  |  |  |  |  | |  |  |  |  | | | |  |
| Zambia |  |  |  |  |  |  |  |  |  |  |  |  |  |  |  |  |  |  |  |  |  |  |  |  |  |  |  |  |  | |  |  |  |  | | | |  |
|  |  |  |  |  |  |  |  |  |  |  |  |  |  |  |  |  |  |  |  |  |  |  |  |  |  |  |  |  |  | |  |  |  |  | | | |  |
|  |  |  |  |  |  |  |  |  |  |  |  |  |  |  |  |  |  |  |  |  |  |  |  |  |  |  |  |  |  | |  |  |  |  | | | |  |

## Supplementary Figure 2. Study timelines indicating the dates of cross-sectional household surveys, months of ANC surveillance data selected for inclusion in models, and dates of ITN distribution campaigns. During the period depicted here, mass ITN campaigns were not conduced in Geita, Tanzania; instead, Geita conducted school-based distribution of ITNs between April and August each year depending on the availability of ITNs.


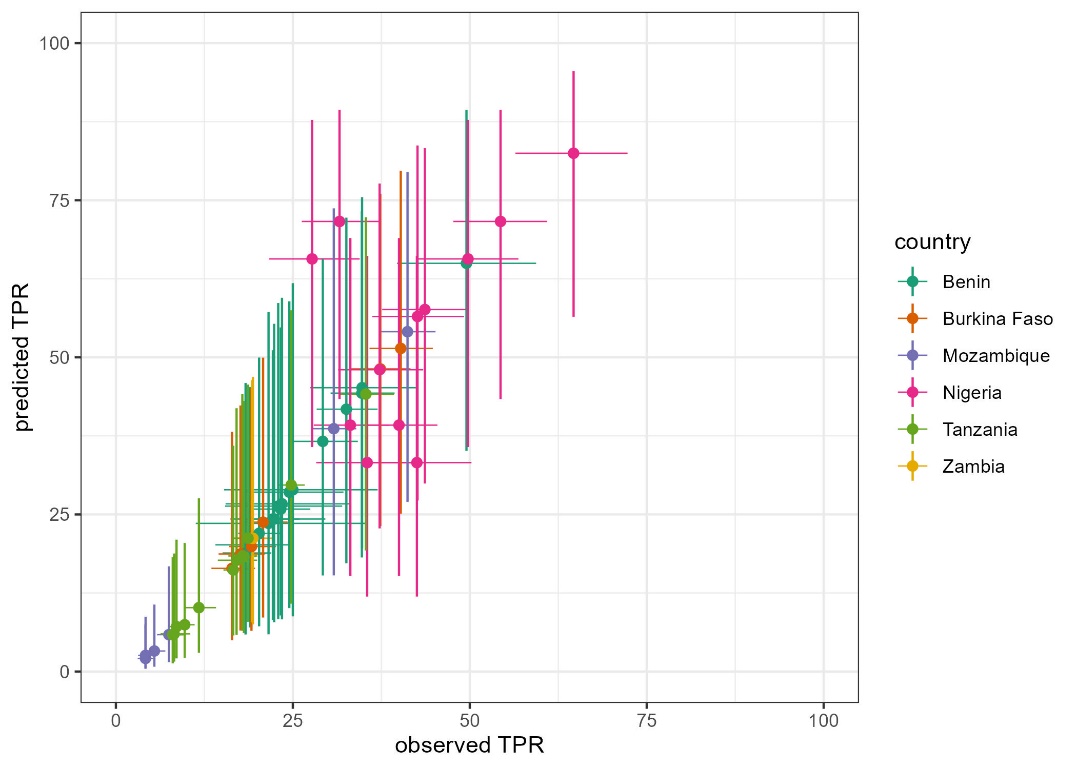


## Supplementary Figure 3a. Predicted vs. observed test positivity rates among children from the leave-one-out cross-validation models using data from all women, colored by country.


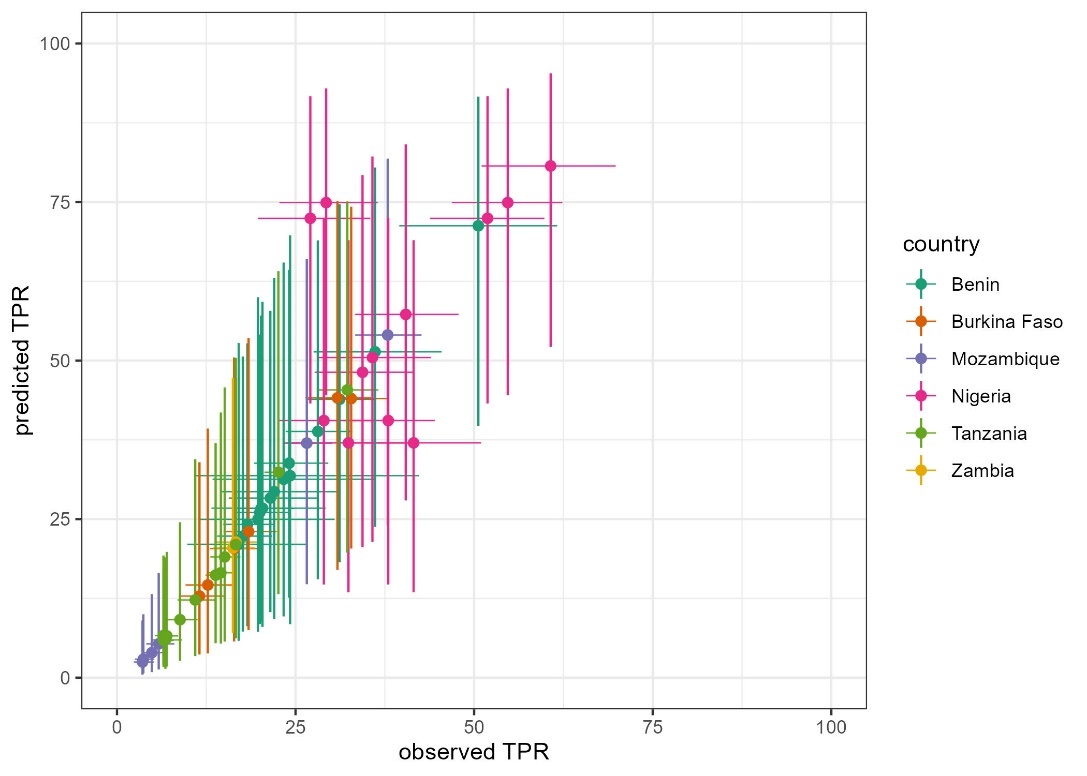


## Supplementary Figure 3b. Predicted vs. observed test positivity rates among children from the leave-one-out cross-validation models using data from multigravida women, colored by country.


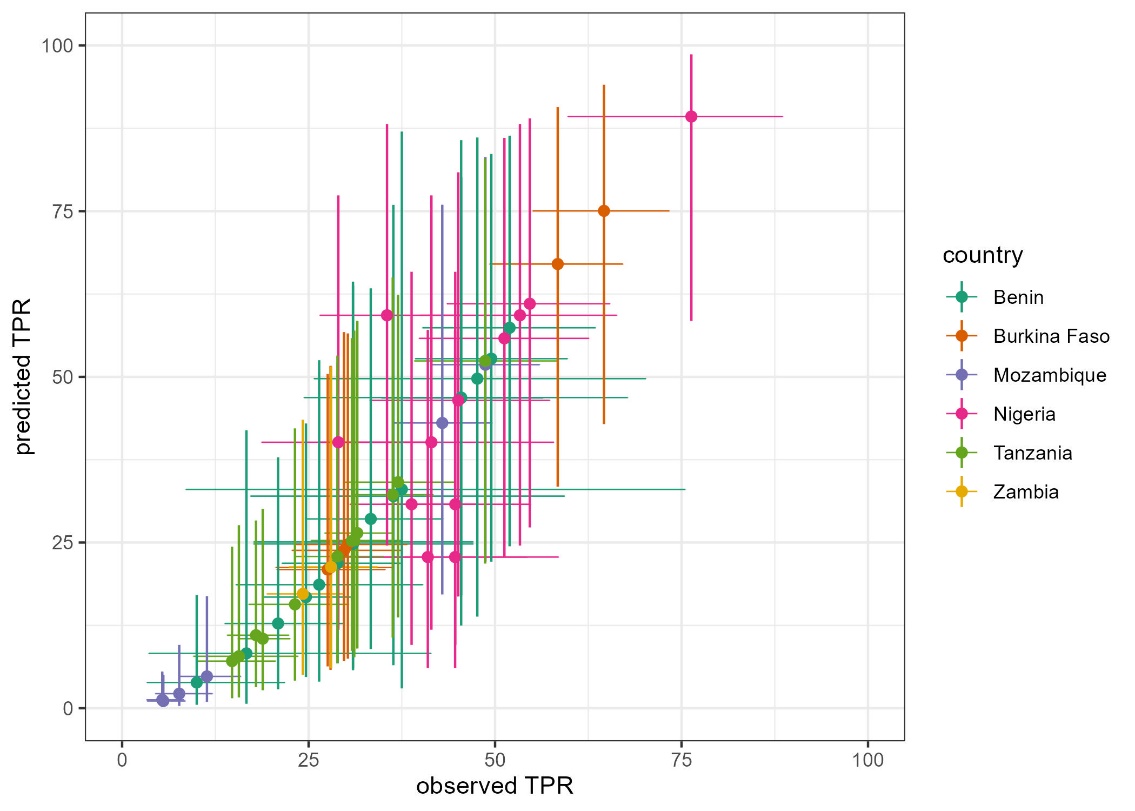


## Supplementary Figure 3c. Predicted vs. observed test positivity rates among children from the leave-one-out cross-validation models using data from primigravid women, colored by country.

## Supplementary Tables

Supplementary Table 1**.** Number of children tested in cross-sectional household surveys and number of ANC1 attendees included in analyses for comparison. Participants have been summed across study years. ANC1 attendees include all gravidae.

| country | district | number of children | number of anc1 attendees (number of health facilities) |
| --- | --- | --- | --- |
| benin | \| Abomey-calavi \| 637 \| 833 (5) \| \| --- \| --- \| --- \| \| Allada \| 922 \| 1,026 (8) \| \| Kpomasse \| 389 \| 278 (3) \| \| Ouidah \| 303 \| 268 (3) \| \| So-ava \| 503 \| 177 (4) \| \| Toffo \| 713 \| 812 (7) \| \| Tori-bossito \| 416 \| 259 (3) \| \| Ze \| 575 \| 968 (5) \| | | |
| burkina faso | \| Banfora \| 396 \| 1,170 (7) \| \| --- \| --- \| --- \| \| Gaoua \| 449 \| 1,012 (7) \| \| Orodara \| 394 \| 1,136 (7) \| | | |
| mozambique | \| Changara \| 843 \| 2,291 (7) \| \| --- \| --- \| --- \| \| Chemba \| 841 \| 1,508 (7) \| \| Guro \| 839 \| 1,903 (7) \| | | |
| nigeria | \| Asa \| 1,358 \| 581 (10) \| \| --- \| --- \| --- \| \| Ejigbo \| 2,059 \| 753 (10) \| \| Ife North \| 2,032 \| 723 (10) \| \| Moro \| 1,453 \| 782 (10) \| | | |
| tanzania | \| Bukombe \| 526 \| 1,515 (3) \| \| --- \| --- \| --- \| \| Chato \| 2,210 \| 4,788 (13) \| \| Geita \| 1,281 \| 3,498 (9) \| \| Geita Town \| 326 \| 3,343 (3) \| \| Mbogwe \| 1,278 \| 3,332 (8) \| \| Nyang’hwale \| 679 \| 1,342 (4) \| | | |
| zambia | \| Chadiza \| 4,020 \| 1,720 (18) \| \| --- \| --- \| --- \| | | |

Supplementary Table 2. Test positivity rates (TPR) and 95% confidence intervals by district, year, and group.

| COUNTRY | district test positivity rate (95% ci) | | | | | | | | | | |
| --- | --- | --- | --- | --- | --- | --- | --- | --- | --- | --- | --- |
|  |  | **2019** | | **2020** | | **2021** | | **2022** | | **2023** | |
|  |  | Household | ANC | Household | ANC | Household | ANC | Household | ANC | Household | ANC |
| Benin | Abomey-calavi |  |  | 18.1  (14-22.7) | 24.5  (17.9-32.2) |  |  | 17.7  (13.7-22.4) | 20.2  (17.3-23.4) |  |  |
|  | Allada |  |  | 32.2  (27.7-37) | 32.6  (28.4-37) |  |  | 42.3  (38-46.7) | 22.2  (18.8-25.9) |  |  |
|  | Kpomasse |  |  | 38.4  (31.1-46.1) | 21.6  (11.3-35.3) |  |  | 47  (40.2-53.9) | 18.9  (14.1-24.7) |  |  |
|  | Ouidah |  |  | 38.5  (30.1-47.4) | 49.5  (39.7-59.4) |  |  | 18.5  (13-25.1) | 34.8  (27.5-42.7) |  |  |
|  | So-ava |  |  | 14.3  (10.1-19.4) | 25  (15.3-37) |  |  | 14.3  (10.4-19.1) | 22.9  (15.4-32) |  |  |
|  | Toffo |  |  | 35.9  (31.1-41) | 34.8  (30.3-39.4) |  |  | 35.6  (30.5-40.9) | 29.2  (24.6-34.2) |  |  |
|  | Tori-bossito |  |  | 29.4  (23.2-36.2) | 23.5  (15.5-33.1) |  |  | 32.1  (25.9-38.8) | 22.4  (16.2-29.6) |  |  |
|  | Ze |  |  | 21  (16.4-26.1) | 18.3  (15.1-21.9) |  |  | 25.4  (20.4-30.8) | 23.3  (19.5-27.5) |  |  |
| Burkina Faso | Banfora |  |  |  |  | 35.5  (28.9-42.6) | 20.8  (17.6-24.3) | 23.1  (17.4-29.6) | 19.2  (16-22.6) |  |  |
|  | Gaoua |  |  |  |  | 50.2  (43.6-56.8) | 37.4  (33.2-41.6) | 69.2  (62.5-75.3) | 40.2  (35.8-44.8) |  |  |
|  | Orodara |  |  |  |  | 19  (13.8-25.1) | 16.4  (13.5-19.7) | 23.7  (17.9-30.3) | 17.6  (14.5-21) |  |  |
| Mozambique | Changara |  |  |  |  | 2.1  (1-4) | 4.2  (3.1-5.5) | 7.6  (5.2-10.5) | 4.2  (3.1-5.5) |  |  |
|  | Chemba |  |  |  |  | 39  (34.3-43.8) | 41.2  (37.3-45.2) | 43.3  (38.5-48.2) | 30.8  (27.7-34) |  |  |
|  | Guro |  |  |  |  | 3.8  (2.2-6.1) | 7.5  (5.8-9.5) | 7.1  (4.9-10) | 5.4  (4.1-7) |  |  |
| Nigeria | Asa |  |  | 63.1  (58.2-67.7) | 64.7  (56.5-72.3) | 41.3  (36.8-45.9) | 54.3  (47.7-60.9) | 47.3  (42.7-51.9) | 49.8  (42.6-56.9) |  |  |
|  | Eligbo |  |  | 38.6  (33.7-43.6) | 37.3  (31.4-43.4) | 34.5  (31.3-37.8) | 31.6  (26.3-37.3) | 42.1  (38.7-45.5) | 27.7  (21.7-34.4) |  |  |
|  | Ife North |  |  | 48.4  (43.3-53.6) | 42.6  (36.2-49.2) | 47.4  (44-50.9) | 33.1  (28-38.6) | 53.1  (49.6-56.6) | 35.5  (28.3-43.2) |  |  |
|  | Moro |  |  | 50.1  (45.2-55) | 43.7  (37.6-49.8) | 30.9  (26.9-35.1) | 40  (34.8-45.4) | 42.7  (38.4-47) | 42.5  (35.1-50.2) |  |  |
| tanzania | Bukombe | 23.2  (18.2-28.8) | 11.7  (9.6-14.1) |  |  | 13.1  (9.3-17.8) | 8.2  (6.3-10.5) |  |  |  |  |
|  | Chato | 22.7  (20.1-25.4) | 16.6  (15.2-18) |  |  | 26.7  (24.2-29.3) | 24.8  (22.9-26.7) |  |  |  |  |
|  | Geita | 33.3  (29.8-37) | 18.1  (16.4-19.8) |  |  | 25.5  (22-29.1) | 18.7  (16.7-20.8) |  |  |  |  |
|  | Geita Town | 3.4  (1.2-7.2) | 8.6  (7.4-9.9) |  |  | 13.6  (8.5-20.2) | 8  (6.7-9.6) |  |  |  |  |
|  | Mbogwe | 17.1  (14.3-20.3) | 9.7  (8.4-11.1) |  |  | 24  (20.8-27.5) | 17.9  (15.9-20) |  |  |  |  |
|  | Nyang’hwale | 8.9  (6.2-12.4) | 17.1  (14.4-20) |  |  | 41.9  (36.5-47.4) | 35.3  (31.5-39.3) |  |  |  |  |
| zambia | Chadiza |  |  |  |  | 21  (19.2-22.8) | 19.4  (16.8-22.1) |  |  | 15.1  (13.6-16.8) | 19.2  (16.6-22.1) |

## Supplementary Tables 3a-d. TPRs by age group, Nigeria, 2021 (3a-b) and 2022 (3c-d).

Supplementary Table 3a. TPRs among children under five, Nigeria, 2021. Children receiving SMC during the current campaign (Asa, Moro districts) were removed from the analysis.

| District | positive | tested | TPR (95% CI) |
| --- | --- | --- | --- |
| Asa | 23 | 41 | 71.5 (39.7 – 71.5) |
| ejigbo | 103 | 412 | 25 (20.9 – 29.5) |
| ife north | 170 | 408 | 41.7 (36.8 – 46.6) |
| moro | 27 | 80 | 33.8 (23.6 – 45.2) |

Supplementary Table 3b. TPRs among children five to fifteen, Nigeria, 2021.

| district | positive | tested | TPR (95% CI) |
| --- | --- | --- | --- |
| Asa | 170 | 426 | 39.9 (35.2 – 44.7) |
| ejigbo | 187 | 428 | 43.7 (38.9 – 48.5) |
| ife north | 225 | 425 | 52.9 (48.1 – 57.8) |
| moro | 130 | 428 | 30.4 (26.0 – 35.0) |

Supplementary Table 3c. TPRs among children under five, Nigeria, 2022. Children receiving SMC during the current campaign (Asa, Moro districts) were removed from the analysis.

| district | positive | tested | TPR (95% CI) |
| --- | --- | --- | --- |
| Asa | 40 | 46 | 87.0 (73.7 – 95.1) |
| ejigbo | 141 | 405 | 34.8 (30.2 – 39.7) |
| ife north | 200 | 402 | 49.8 (44.8 – 54.7) |
| moro | 52 | 114 | 45.6 (36.3 – 55.2) |

Supplementary Table 3d. TPRs among children five to fifteen, Nigeria, 2022.

| district | positive | tested | TPR (95% CI) |
| --- | --- | --- | --- |
| Asa | 184 | 428 | 43.0 (38.2 – 47.8) |
| ejigbo | 206 | 420 | 49.0 (44.2 – 53.9) |
| ife north | 235 | 417 | 56.4 (51.4 – 61.2) |
| moro | 175 | 418 | 41.9 (37.1 – 46.8) |

## Supplementary Tables 4a-d. TPRs by age group, Burkina Faso, 2021 (4a-b) and 2022 (4c-d).

Supplementary Table 4a. TPRs among children under five, Burkina Faso, 2021. Children receiving SMC during the current campaign were removed from the analysis.

| district | positive | tested | TPR (95% CI) |
| --- | --- | --- | --- |
| Banfora | 1 | 6 | 16.7 (0.4 – 64.1%) |
| gaoua | 14 | 44 | 31.8 (18.6 – 47.6%) |
| orodara | 0 | 9 | 0 (0 – 33.6%) |

Supplementary Table 4b. TPRs among children five to ten, Burkina Faso, 2021.

| district | positive | tested | TPR (95% CI) |
| --- | --- | --- | --- |
| Banfora | 69 | 191 | 36.1 (29.3 – 43.4%) |
| gaoua | 104 | 191 | 54.4 (47.1 – 61.7%) |
| orodara | 38 | 191 | 19.9 (14.5 – 26.3%) |

Supplementary Table 4c. TPRs among children under five years, Burkina Faso, 2022. Children receiving SMC during the current campaign were removed from the analysis.

| district | positive | tested | TPR (95% CI) |
| --- | --- | --- | --- |
| Banfora | 3 | 8 | 37.5 (8.5 – 75.5%) |
| gaoua | 6 | 22 | 27.3 (10.7 – 50.2%) |
| orodara | 1 | 3 | 33.3 (0.8 – 90.6%) |

Supplementary Table 4d. TPRs among children five to ten years, Burkina Faso, 2022.

| district | positive | tested | TPR (95% CI) |
| --- | --- | --- | --- |
| Banfora | 43 | 191 | 22.5 (16.8 – 29.1%) |
| gaoua | 142 | 192 | 74.0 (67.1 – 80.0%) |
| orodara | 45 | 191 | 23.6 (17.7 – 30.2%) |

Supplementary Table 5. ITN ownership at the level of one net per household (95% confidence intervals) by district, year, and group.

|  |  |  |  |  |  |  |  |  |  |
| --- | --- | --- | --- | --- | --- | --- | --- | --- | --- |
| country | **DISTRICT** | **% REPORTING OWNERSHIP OF AT LEAST ONE ITN (95% CI)** | | | | | | | |
|  |  | **2020** | | **2021** | | **2022** | | **2023** | |
|  |  | Household | ANC | Household | ANC | Household | ANC | Household | ANC |
| Benin | Allada | 97.1  (95.3-98.4) | 99.3  (96.2-100) |  |  | 91.2  (88.4-93.5) | 95.3  (93.4-96.7) |  |  |
|  | Kpomasse | 96.8  (93.1-98.8) | 100  (76.8-100) |  |  | 92.5  (87.7-95.8) | 92.4  (87.4-95.9) |  |  |
|  | Ouidah | 97.3  (93.8-99.1) | 96.8  (83.3-99.9) |  |  | 94  (89.6-97) | 94.8  (90.4-97.6) |  |  |
|  | So-ava | - | - |  |  | 99.2  (97.1-99.9) | 96.5  (95-97.6) |  |  |
|  | Toffo | 97  (94.9-98.4) | 93  (87.5-96.6) |  |  | 94  (91.3-96) | 94.8  (92.4-96.6) |  |  |
|  | Tori-bossito | 94.3  (90.6-96.9) | 96.4  (81.7-99.9) |  |  | 82.3  (76.9-86.8) | 86.2  (81.4-90.2) |  |  |
|  | Ze | 97.4  (95-98.9) | 98.3  (95.2-99.7) |  |  | 90.1  (86.2-93.1) | 92.3  (89.5-94.6) |  |  |
| Burkina Faso | Banfora |  |  | 96.3  (92.6-98.5) | 84.8  (81.1-88) | 80  (73.6-85.4) | 79.4  (75.1-83.2) |  |  |
|  | Gaoua |  |  | 56.8  (49.5-64) | 51.4  (46.3-56.5) | 50.5  (43.2-57.8) | 44.1  (38.4-50) |  |  |
|  | Orodara |  |  | 99.5  (97.1-100) | 74.3  (69.3-78.8) | 98.9  (96.2-99.9) | 80  (75.4-84.1) |  |  |
| Mozambique | Changara |  |  | 96.2  (93.9-97.8) | 92.6  (90.7-94.2) | 48  (43.1-52.9) | 82.4  (79.3-85.2) |  |  |
|  | Chemba |  |  | 98.8  (97.3-99.6) | 92  (89.1-94.4) | 91.1  (88-93.7) | 88.2  (85.1-90.9) |  |  |
|  | Guro |  |  | 98.3  (96.6-99.3) | 90.3  (87.2-93) | 76  (71.6-80) | 57.9  (54.1-61.7) |  |  |
| Nigeria | Asa |  |  | 66.4  (61.7-70.9) | 92  (87.3-95.3) | 67.1  (62.3-71.5) | 91.3  (86-95) |  |  |
|  | Eligbo |  |  | 76  (71.6-80) | 71.8  (65-77.9) | 56.1  (51.1-60.9) | 28.1  (21.9-35.1) |  |  |
|  | Ife North |  |  | 67.5  (62.8-72) | 92  (87.7-95.2) | 75.2  (70.8-79.3) | 94.2  (89.6-97.2) |  |  |
|  | Moro |  |  | 51.1  (46.2-56) | 53.3  (47.2-59.4) | 33  (28.4-37.8) | 22.9  (16.5-30.4) |  |  |
| zambia | Chadiza |  |  | 57.9  (55.9-59.9) | 75.4  (72.4-78.2) |  |  | 54.7  (52.7-56.6) | 56.7  (53.3-60.1) |

Supplementary Table 6. ITN ownership at the level of one net per two persons (95% confidence intervals) by district, year, and group.

|  |  |  |  |  |  |  |  |  |  |
| --- | --- | --- | --- | --- | --- | --- | --- | --- | --- |
| country | **DISTRICT** | **% REPORTING OWNERSHIP OF AT LEAST ONE ITN PER TWO PERSONS (95% CI)** | | | | | | | |
|  |  | **2020** | | **2021** | | **2022** | | **2023** | |
|  |  | Household | ANC | Household | ANC | Household | ANC | Household | ANC |
| Benin | Allada | 51.1  (46.6-55.6) | 67.1  (58.9-74.7) |  |  | 32.4  (28.3-36.7) | 53.3  (49.4-57.1) |  |  |
|  | Kpomasse | 53.5  (46.1-60.8) | 57.1  (28.9-82.3) |  |  | 50.5  (43.1-57.9) | 50  (42.3-57.7) |  |  |
|  | Ouidah | 51.4  (43.9-58.8) | 71  (52-85.8) |  |  | 40.2  (33.1-47.7) | 49.7  (42-57.4) |  |  |
|  | So-ava | - | - |  |  | 42.7  (36.4-49.1) | 47.8  (44.4-51.2) |  |  |
|  | Toffo | 48.7  (43.9-53.5) | 55.2  (46.7-63.6) |  |  | 37.2  (32.6-41.9) | 47.8  (43.2-52.5) |  |  |
|  | Tori-bossito | 48.8  (42.4-55.2) | 57.1  (37.2-75.5) |  |  | 28.6  (23.1-34.7) | 40.9  (34.8-47.3) |  |  |
|  | Ze | 43.4  (37.8-49.1) | 63.3  (55.8-70.4) |  |  | 30.1  (25.1-35.6) | 49.4  (44.7-54) |  |  |
| Burkina Faso | Banfora |  |  | 38.4  (31.5-45.7) | 49.9  (45.2-54.6) | 36.8  (30-44.1) | 44.2  (39.3-49.2) |  |  |
|  | Gaoua |  |  | 16.3  (11.4-22.4) | 23.1  (19-27.7) | 15.8  (10.9-21.8) | 16.4  (12.4-21.1) |  |  |
|  | Orodara |  |  | 51.3  (44-58.6) | 40.5  (35.3-46) | 54.7  (47.4-62) | 55.9  (50.5-61.3) |  |  |
| Mozambique | Changara |  |  | 59.8  (54.9-64.5) | 64.4  (61.2-67.6) | 11.1  (8.3-14.5) | 52.9  (49.1-56.8) |  |  |
|  | Chemba |  |  | 61.8  (56.9-66.4) | 44.7  (39.9-49.6) | 43.1  (38.3-48) | 30.4  (26.4-34.6) |  |  |
|  | Guro |  |  | 63.7  (58.9-68.3) | 46.9  (42.1-51.7) | 33.8  (29.3-38.6) | 26.6  (23.4-30.1) |  |  |
| Nigeria | Asa |  |  | 15.4  (12.1-19.2) | 54.8  (47.6-61.8) | 17.4  (13.9-21.4) | 37.8  (30.5-45.5) |  |  |
|  | Eligbo |  |  | 29.4  (25.1-34) | 53.5  (46.3-60.5) | 18.9  (15.3-23.1) | 24  (18.1-30.6) |  |  |
|  | Ife North |  |  | 28.9  (24.6-33.5) | 80.9  (75.1-85.8) | 36.4  (31.8-41.3) | 82  (75.4-87.4) |  |  |
|  | Moro |  |  | 8  (5.6-11) | 32.2  (26.7-38.2) | 6.2  (4-9) | 13.7  (8.7-20.2) |  |  |
| zambia | Chadiza |  |  | 20  (18.4-21.7) | 25.5  (22.7-28.5) |  |  | 15.5  (14-16.9) | 18.4  (15.9-21.2) |

|  | Setting | % children under 5 with fever  (# fevers/# children under 5) | % sought treatment for child with fever (# sought treatment/# fevers with data) | % tested among those who sought treatment (# tested/# sought treatment) | % received any medication among those who sought treatment (# received medication/# sought treatment) |
| --- | --- | --- | --- | --- | --- |
| Benin | ANC | 10.1  (2,267/22,490) | 66  (705/1,068) | 51.8  (357/689) | 97.7  (599/613) |
|  | HH | 21.8  (1,332/6,121) | 61.5  (643/1,045) | 30.1  (170/551) | 89.1  (481/540) |
| Burkina Faso | ANC | 12.7  (1,074/8,489) | 80.1  (860/1,074) | 88.3  (733/830) | 77.8  (661/850) |
|  | HH | 2.6  (39/1,496) | 52.8  (38/72) | 71.1  (27/38) | 86.5  (32/37) |
| Mozambique | ANC | 7.6  (1,213/15,962) | 86.2  (1,046/1,213) | 87.2%  (898/1,030) | 96.7  (1,000/1,034) |
|  | HH | 4.6  (449/9,840) | 66.3  (63/95) | 65.1  (41/63) | 79.4  (50/63) |
| Nigeria | ANC | 8.9  (789/8,823) | 95.6  (745/779) | 75.6  (563/745) | 98.7  (735/745) |
|  | HH | 1.1  (46/4,334) | 47.9  (23/48) | 69.6  (16/23) | 82.6  (19/23) |
| Tanzania | ANC | 3.8  (787/20,449) | 82.9  (1,946/2,348) | 78.9  (1,535/1,946) | 98.5  (1,917/1,946) |
|  | HH | 8.7  (556/6,393) | 72.3  (633/875) | 47.7  (302/633) | 89.4  (566/633) |
| Zambia | ANC | 30.8  (2,019/6,558) | 93.7  (610/651) | 94.4  (576/610) | 93.9  (573/610) |
|  | HH | 12.5  (508/4,077) | 68.8  (327/475) | 100  (289/289) | 88.7  (289/326) |

Supplementary Table 7. Data on children under 5 years with fever, treatment-seeking, testing and treatment among those who sought treatment by setting (ANC or HH) and country.
